# Biological evaluation of pyrazolyl-urea and dihydro-imidazo-pyrazolyl-urea derivatives as potential anti-angiogenetic agents in the treatment of neuroblastoma

**DOI:** 10.18632/oncotarget.27733

**Published:** 2020-09-15

**Authors:** Barbara Marengo, Elda Meta, Chiara Brullo, Chiara De Ciucis, Renata Colla, Andrea Speciale, Ombretta Garbarino, Olga Bruno, Cinzia Domenicotti

**Affiliations:** ^1^Department of Experimental Medicine, General Pathology Section, University of Genoa, Genoa, Italy; ^2^Department of Pharmacy, Medicinal Chemistry Section, University of Genoa, Genoa, Italy; ^3^Department of Oncology, Laboratory of Angiogenesis and Vascular Metabolism, Katholieke Universiteit Leuven, Leuven, Belgium; ^4^Laboratory of Angiogenesis and Vascular Metabolism, Center for Cancer Biology, Vlaams Instituut voor Biotechnologie, Leuven, Belgium; ^5^UOC Mutagenesis and Cancer Prevention, IRCCS Ospedale Policlinico San Martino, Genoa, Italy; ^*^These authors contributed equally to this work

**Keywords:** tumor angiogenesis, neuroblastoma, vascular mimicry, pyrazolyl-urea derivatives, dihydro-imidazo-pyrazolyl-urea derivatives

## Abstract

Pyrazolyl-urea and dihydro-imidazo-pyrazolyl-urea compounds (STIRUR 13, STIRUR 41 and BUR 12) have been demonstrated to exert a strong inhibitory effect on interleukin 8 or N-formyl-methionyl-leucyl-phenylalanine-induced chemotaxis of human neutrophils. Since the migration of cancer cells is comparable to that of neutrophils, the purpose of this study is to evaluate the biological effect of STIRUR 13, STIRUR 41 and BUR 12 on ACN and HTLA-230, two neuroblastoma (NB) cell lines with different degree of malignancy. HTLA-230 cells, stage-IV NB cells, have high plasticity and can serve as progenitors of endothelial cells. The results herein reported show that the three tested compounds were not cytotoxic for both NB cells and did not alter their clonogenic potential. However, all compounds were able to inhibit the ability of HTLA-230 to form vascular-like structures. On the basis of these findings, pyrazolyl-urea and dihydro-imidazo-pyrazolyl-urea derivatives could be proposed as agents potentially effective in counteracting NB malignancy by inhibiting cell migration and tumor angiogenesis which represent important hallmarks responsible for cancer survival and progression.

## INTRODUCTION

**Figure 1:**
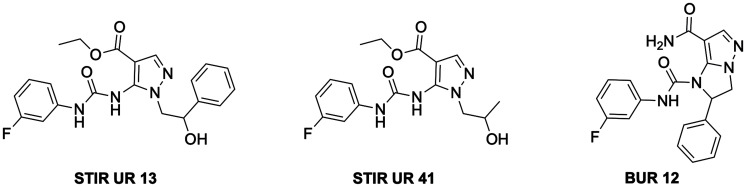
Chemical structure of STIRUR 13, STIRUR 41 and BUR 12.

Chemotaxis is a complex process which consists of neutrophil migration to the site of inflammation and it is regulated by downstream signaling molecules, including phosphatidylinositol 3-kinase (PI3K), phospholipase C, mitogen-activated protein kinases (MAPKs), and extracellular response kinases 1 and 2 (ERK1/2). In this context, it has been previously demonstrated that pyrazolyl-urea and dihydro-imidazo-pyrazolyl-urea compounds, namely STIRUR 13, STIRUR 41 and BUR 12, have a strong inhibitory effect on interleukin-8 (IL-8) or N-formyl-methionyl-leucyl-phenylalanine (fMLP)-induced chemotaxis of human neutrophils [1–3]. The chemical structure of STIRUR 13, STIRUR 41 and BUR 12, that we have previously synthesized [1, 3], is reported in Figure 1. The three molecules have been chosen from the large library of chemotaxis inhibitors, because they showed a different biological profile. In detail, STIRUR 13 inhibited only IL-8 induced chemotaxis, whereas STIRUR 41 and BUR 12 were able to block both IL8-induced and fMLP-induced neutrophil migration, even though with a different degree of biological activity. As concerns the chemical structure, STIRUR 13 and STIRUR 41 are both 3-fluoro-phenyl-5-pyrazolyl-urea derivatives that differ on the basis of the chain in N1 position whereas, BUR 12 is a dihydro-imidazo-pyrazole characterized by a more rigid scaffold that was obtained by a condensation of OH and NH_2_ functional groups present in the pyrazole derivatives and it can be considered a constrained form of STIRUR 13 in which the urea group is partially inserted in the imidazole core.

Both STIRUR derivatives have a carboxymethyl function in position 4 while BUR12 bears a carboxyamide group in position 7, that resulted stable under all chemical manipulations. Although the presence of a carboxylic group (obtained by a hydrolyzing reaction) is excluded in the conditions of *in vitro* studies here reported, it is not possible to exclude the generation of active metabolites *in vivo*.

Moreover, it is important to note that all compounds have a 3-fluorophenyl substituent on the urea moiety, which probably confers a greater lipophilicity. In fact, logP analysis performed by ACD Labs software showed that STIRUR13, STIRUR41 and BUR12 had clog*P* values of 5.19, 3.74 and 1.48 respectively. The same analysis performed on their analogues, lacking the fluoro substituent in the phenyl-urea moiety, reported, as expected, lower clog*P* values (4.74, 3.25 and 0.99, respectively), confirming that the fluorine atom increases the lipophilicity of the molecules. STIRUR13 resulted more lipophilic than STIRUR41 for the presence of a phenyl ring instead of a methyl group to the terminal chain in N1 position. The lower clogP of BUR12, despite the lack of the polar hydroxy group in the same chain, is probably due to the amide function in position 7. However, all compounds showed clog*P* values major than 1, and therefore they could have the pharmacokinetic properties necessary to permeate cell membranes.

Interestingly, among the pyrazole and imidazopyrazole derivatives, that we have previously synthesized, the compounds having a 3-fluoro-phenyl substituent always showed a better pharmacological profile.

Recently, these compounds have been demonstrated to reduce neutrophil chemotaxis and movement by inhibiting the activity of both p38MAPK and classic protein kinase C (PKC) isoforms without affecting the activity of novel PKC isoenzymes and of protein kinase B (PKB)/Akt [4]. However, it has been reported that the activation of p38MAPK is also involved in the migration and invasiveness of high-risk human neuroblastoma cells [5]. Interestingly, relapsed and refractory neuroblastoma contains mutations that are predicted to activate Ras/MAPK signaling [6], and the ability of MAPK inhibitors to inhibit neuroblastoma growth has been demonstrated both *in vitro* and *in vivo* [7].

Neuroblastoma (NB) is an aggressive paediatric tumor that is able to produce high levels of chemokines [8, 9] facilitating the movement of neutrophils to the tumor site. Among chemokines, IL-8 (CXCL8) stimulates the migration of neutrophils to the site of inflammation and also the migration of cancer cells facilitating the metastatic process [10, 11]. Moreover, IL-8 is highly expressed by several human cancers and it plays an important role in angiogenesis as well as in vascular mimicry [12–15], where cancer cells mimic endothelial cells and form blood vessels [13, 14, 16]. Interestingly, it has been shown that HTLA-230 cells, stage-IV NB cells with MYCN amplification, have high plasticity and can serve as progenitors of endothelial cells [17, 18]. It is important to note that MYCN amplification has been widely recognized to be a strong prognostic indicator of poor prognosis, and also the best-characterized genetic marker for NB stratification [19, 20]. Furthermore, MYCN amplification has been reported to correlate with a high tumor vascularity [21] and it has been demonstrated that NB expresses an up-regulation of macrophage migration inhibitory factor (MIF) which is able to promote the expression of MYCN, *via* ERK activity, and the secretion of IL-8 and of angiogenic factors [22].

Therefore, given that the movement of malignant cancer cells can be compared to that of migrant neutrophils the aim of this study was to analyze the biological effect of STIRUR 13, STIRUR 41 and BUR 12 on NB cell lines characterized by a different status of MYCN amplification.

The results herein reported suggest that these new compounds could be promising potential drugs able to counteract high risk-NB recurrence and relapse by counteracting inflammation and angiogenesis, two mechanisms strongly involved in cancer progression and metastatization.

## RESULTS

### STIRUR 13, STIRUR 41 and BUR 12 treatment do not affect the viability and tumorigenicity of ACN and HTLA-230 cells

**Figure 2:**
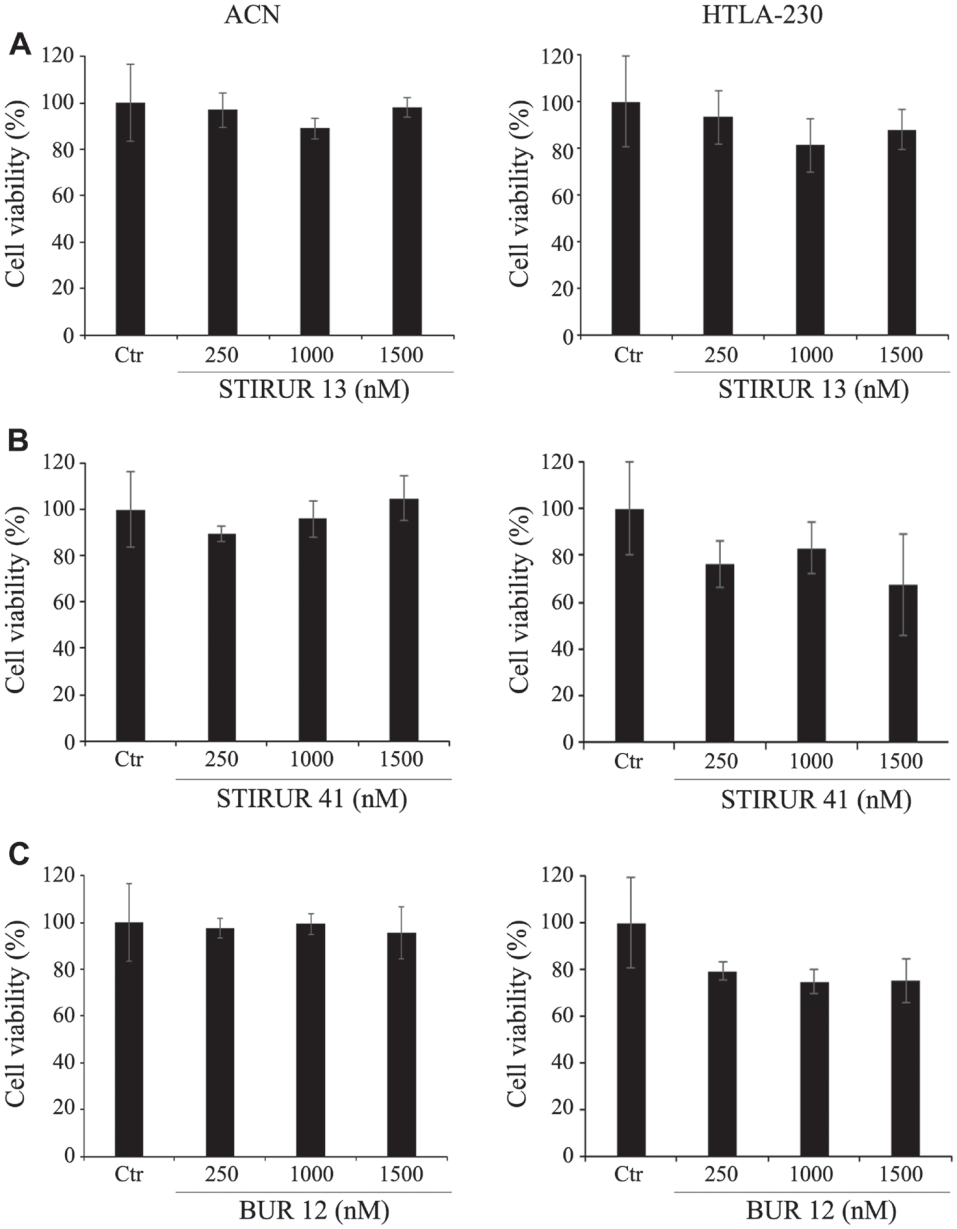
STIRUR 13, STIRUR 41 and BUR 12 did not affect cell viability of ACN and HTLA-230 cells. Cell viability was determined by MTT assays. ACN cells (left panels) and HTLA-230 (right panels) cells were exposed to increasing concentrations (250, 1000 and 1500 nM) of STIRUR 13 (**A**) or STIRUR 41 (**B**) or BUR 12 (**C**) for 24 h. Histograms summarize quantitative data of the means ± S. E. M. of four independent experiments.

The biological effects of the pyrazolyl-ureas and dihydro-imidazo-pyrazolyl-ureas (STIRUR 13, STIRUR 41 and BUR 12) were tested on two NB cell lines with a different status of MYCN oncogene activation, namely ACN (without MYCN amplification) and HTLA-230 (with MYCN amplification) cells. As shown in Figure 2, after 24 h treatment, none of the three compounds, STIRUR 13 (A), STIRUR 41 (B) or BUR 12 (C) at the tested doses (250, 1000 and 1500 nM), induced significant alterations of ACN (left panels) or HTLA-230 (right panels) cell viability.

**Figure 3:**
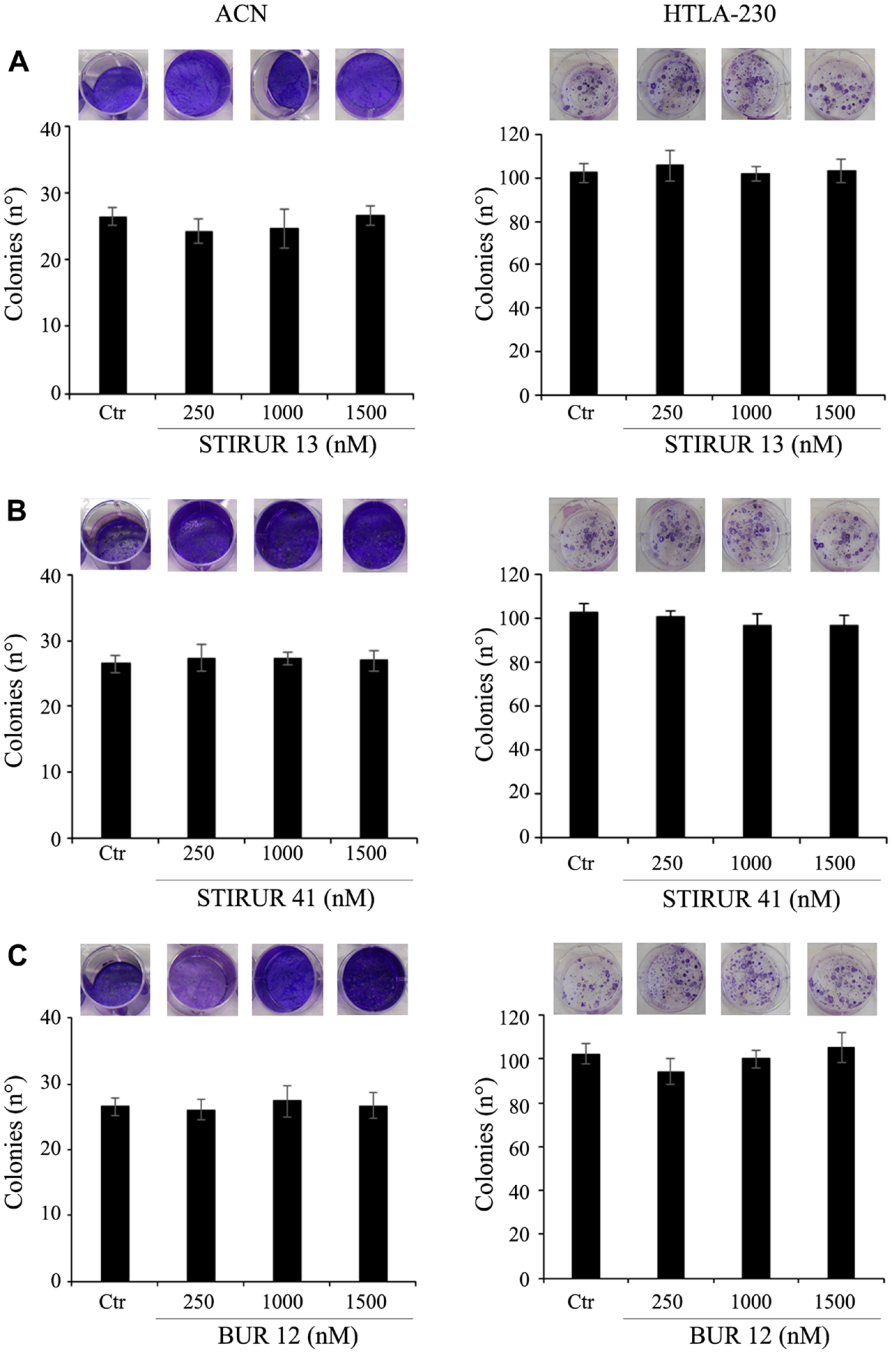
STIRUR 13, STIRUR 41 and BUR 12 did not affect the clonogenic potential of ACN and HTLA-230 cells. The clonogenic potential of ACN (left panels) and HTLA-230 (right panels) cells was evaluated by soft-agar colony formation assay and clonogenic assay, respectively. ACN cells were seeded in 6-well plates and then treated with 250, 1000 and 1500 nM of STIRUR 13 (**A**), STIRUR 41 (**B**) or BUR 12 (**C**) for 24 h, washed and re-plated in agar-containing medium. After 25 days, colonies were stained and counted. HTLA-230-cells were seeded in 6-well plates and then incubated with 250, 1000 and 1500 nM of STIRUR 13 (A), STIRUR 41 (B) or BUR 12 (C) for 24 h. Subsequently, cells were incubated in fresh medium without the drug for an additional 20 days before staining and counting the colonies. Histograms summarize quantitative data of the means ± S. E. M. of four independent experiments.

The effects of the three compounds on tumorigenicity were evaluated by the clonogenic assay in HTLA-230 cells (Figure 3, right panels) [23] while, for ACN cells, an anchorage-independent growth assay was used (Figure 3, left panels) as they were unable to form colonies with the clonogenic assay (data not shown). As shown in Figure 3, untreated ACN and HTLA-230 cells were able to form colonies. However, 24 h exposure of both NB cell lines to STIRUR 13 (Figure 3A), STIRUR 41 (Figure 3B) and BUR 12 (Figure 3C) did not influence the formation of colonies, supporting the evidence that these compounds were not cytotoxic, as previously observed in neutrophils [1–3]. Since these derivatives were reported to limit neutrophil chemotaxis, their capability to interfere with cancer cell migration was investigated.

### STIRUR 13, STIRUR 41 and BUR 12 differently affect the migratory ability of ACN and HTLA-230 cells

**Figure 4:**
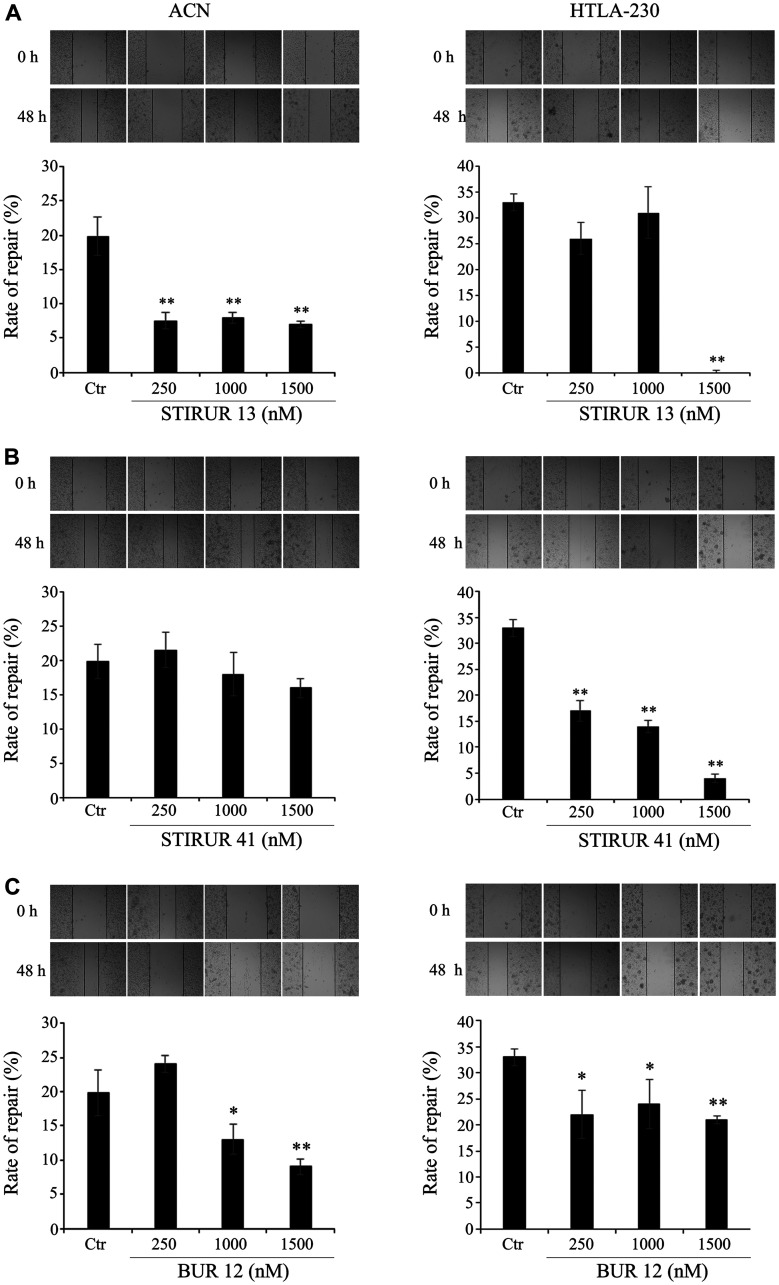
STIRUR 13, STIRUR 41 and BUR 12 affect the scratch repair ability of ACN and HTLA-230 cells. The ability of cells to heal the wound was evaluated by the scratch assay. Representative images of ACN (left panels) and HTLA-230 cells (right panels) scratch assays. The images were taken immediately after the scratch formation and after 48 h. Black lines indicate the wound borders at the beginning of the assay (0 h) and after 48 h. The rate of repair, an indirect method to measure cell migration, was quantified by measuring the distance between the migrating cell boundaries. ACN (left panels) and HTLA-230 (right panels) cells were seeded in 24-well plates and then treated with 250, 1000 and 1500 nM of STIRUR 13 (**A**), STIRUR 41 (**B**) or BUR 12 (**C**) for 24 h. Histograms summarize quantitative data of means ± S. D. of three independent experiments. ^*^ *p* < 0.05 vs control cells; ^**^ *p* < 0.01 vs control cells.

As shown in Figure 4 (left panels), untreated ACN cells at 48 h after the scratch formation, were able to reduce the amplitude of the wound by 20% and the treatments with the three compounds affected the repair ability of ACN cells differently. In particular, STIRUR 13 was able to efficiently counteract the migratory ability of untreated cells at all tested concentrations (Figure 4A, left panel) whereas BUR 12 induced a similar effect but only at the highest concentrations (Figure 4C, left panels). Instead, STIRUR 41 exerted no effect on the repair activity of ACN cells at any of the tested doses (Figure 4B, left panel). In comparison to ACN cells, untreated HTLA-230 cells had a major ability to repair the scratch reducing the wound amplitude by 32% (Figure 4, right panels). STIRUR 13 was able to totally inhibit the repair ability of HTLA-230 at the highest dose (1500 nM; Figure 4A, right panel) while STIRUR 41 induced a reduction of the scratch repair in a dose-dependent manner (Figure 4B, right panel). Instead, BUR 12 reduced the amplitude of the wound by about 30% at all tested doses in respect to untreated cells (Figure 4C, right panel).

**Figure 5:**
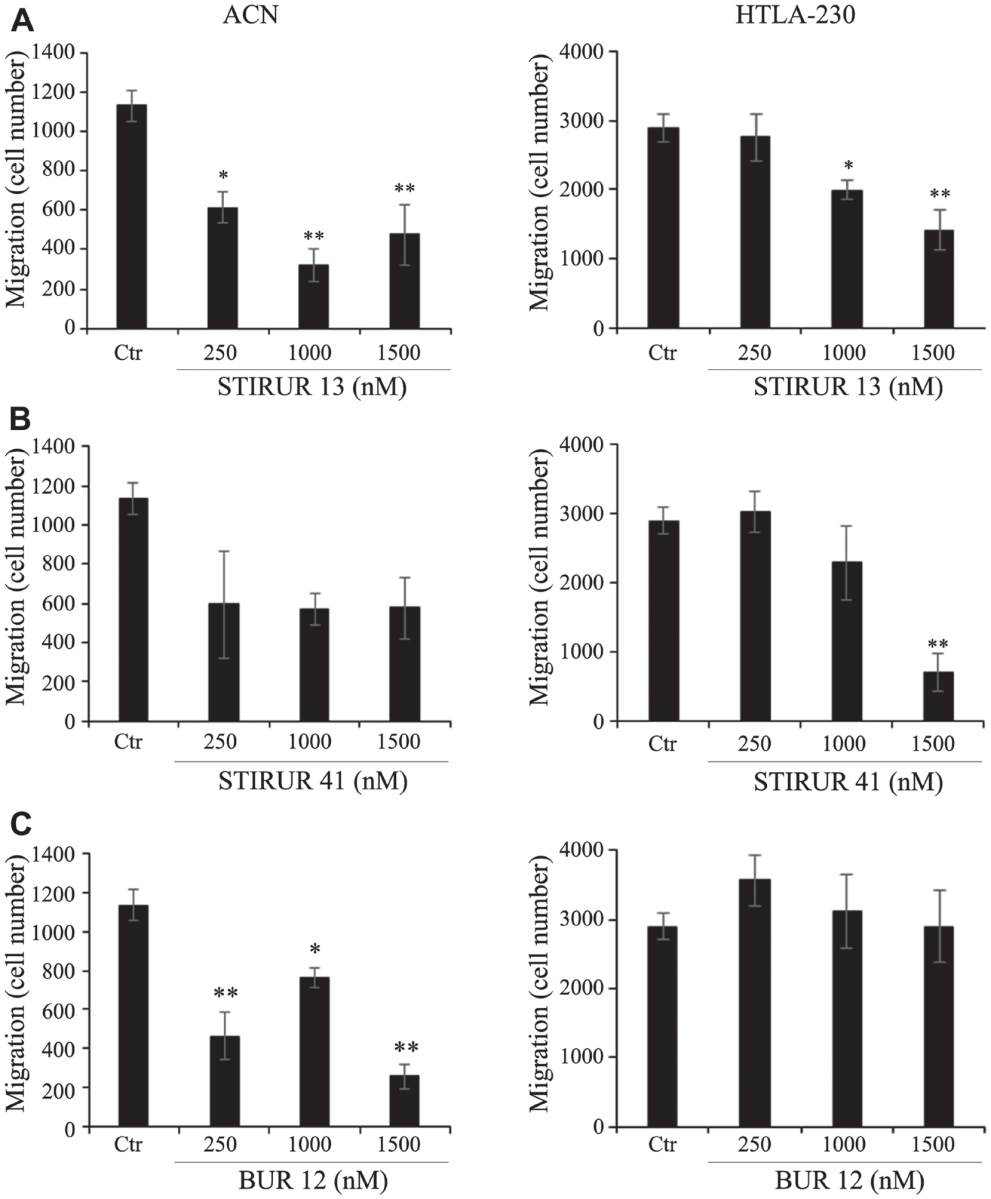
Effects of STIRUR 13, STIRUR 41 and BUR 12 on the migration of ACN and HTLA-230 cells. Cell migration was evaluated by the Transwell assay and quantified by counting the number of cells which moved to the underside of the membrane after 24 h of treatment. ACN (left panels) and HTLA-230 (right panels) cells were seeded in 24-well plates and then treated with 250, 1000 and 1500 nM of STIRUR 13 (**A**), STIRUR 41 (**B**) or BUR 12 (**C**) for 24 h. Histograms summarize quantitative data of means ± S. D. of three independent experiments. ^*^ *p* < 0.05 vs control cells; ^**^ *p* < 0.01 vs control cells.

ACN cells had a minor migratory ability compared to HTLA-230 (Figure 5, left and right panels) as shown by the analysis of the number of ACN cells (approx. 1200) and HTLA-230 cells (approx. 3000) that moved across the polycarbonate filter. The most significant inhibitory effects on ACN cell migration were observed at 1000 and 1500 nM STIRUR 13 (Figure 5A, left panel) and at 1500 nM BUR 12, with a reduction of the migration by about 85% in respect to untreated cells (Figure 5C, left panel). No significant effect on ACN migratory ability was observed at any of the STIRUR 41 tested doses (Figure 5B, left panel). In HTLA-230, STIRUR 13 decreased cell migration by 59% at the highest dose tested (1500 nM; Figure 5A, right panel) and 80% reduction of the migratory ability was observed in HTLA-230 cells treated with the highest concentration of STIRUR 41 (1500 nM; Figure 5B, right panel). However, BUR 12 did not alter cell migration at any of the tested doses (Figure 5B, right panel).

### STIRUR 13, STIRUR 41 and BUR 12 do not induce changes in p38MAPK and phospho-p38MAPK levels of ACN and HTLA-230 cells

Given that p38MAPK inhibitors have been demonstrated to influence the migration of human neuroblastoma cells [5], the effects of the three compounds tested on p38MAPK and phospho-p38MAPK levels was investigated.

**Figure 6:**
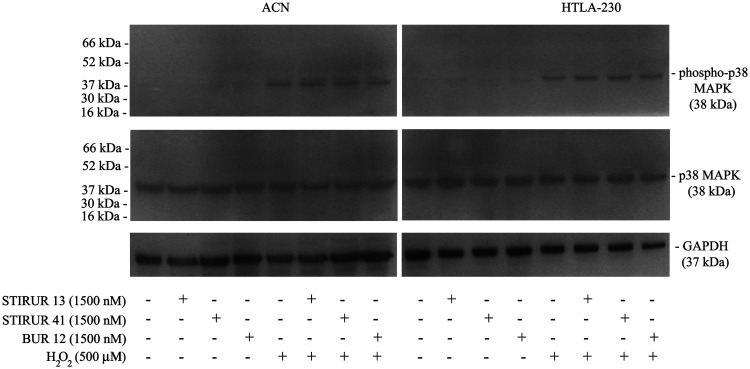
Effects of STIRUR 13, STIRUR 41 and BUR 12 on p38MAPK activation in ACN and HTLA-230 cells. p38MAPK activation was evaluated by immunoblot analyses of phospho-p38MAPK and total p38MAPK. p38MAPK and phospho-p38MAPK expression levels were evaluated in both NB cells treated with STIRUR 13, STIRUR 41, BUR12 (1500 nM), for 24 h, co-treated with H_2_O_2_ (500 μM) after 19 h up to 24 h and treated with H_2_O_2_ (500 μM) for 5 h. GAPDH is the internal loading control.

As shown in Figure 6, 24 h treatment with STIRUR 13, STIRUR 41 or BUR 12 at the highest dose tested (1500 nM), did not induce alterations of p38MAPK and phospho-p38MAPK levels both in ACN (left panels) and HTLA-230 (right panels) cells. However, the kinase was able to be activated either in ACN (left panels) and in HTLA-230 (right panels) cells treated with H_2_O_2_ (500 μM) for 5 h and co-treated with H_2_O_2_ and STIRUR 13, STIRUR 41 or BUR 12 (Figure 6). It is important to note that p38MAPK activation observed in co-treated NB cells was due to H_2_O_2_-induced effect.

### STIRUR 13, STIRUR 41 and BUR 12 counteract the ability of HTLA-230 cells to form capillary-like structures

**Figure 7:**
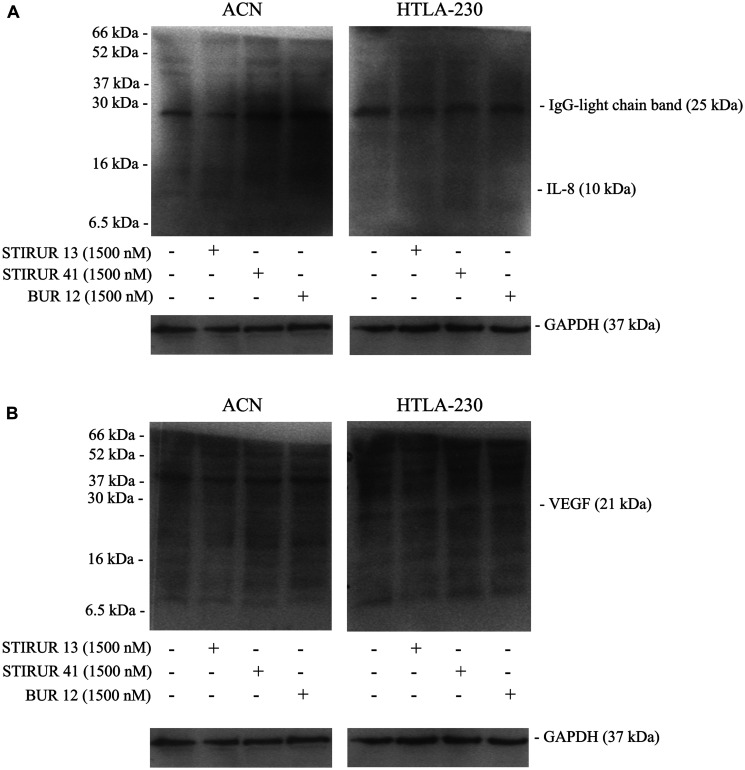
ACN and HTLA-230 do not express IL-8 and only HTLA-230 cells express VEGF. IL-8 (**A**) and VEGF (**B**) expression levels were evaluated in NB cells treated for 24 h with STIRUR 13, STIRUR 41, BUR12 (1500 nM). GAPDH is the internal loading control. The presence of the band corresponding to the IgG-light chain (25 kDa) demonstrates the reactivity of the antibody used to detect IL-8.

**Figure 8:**
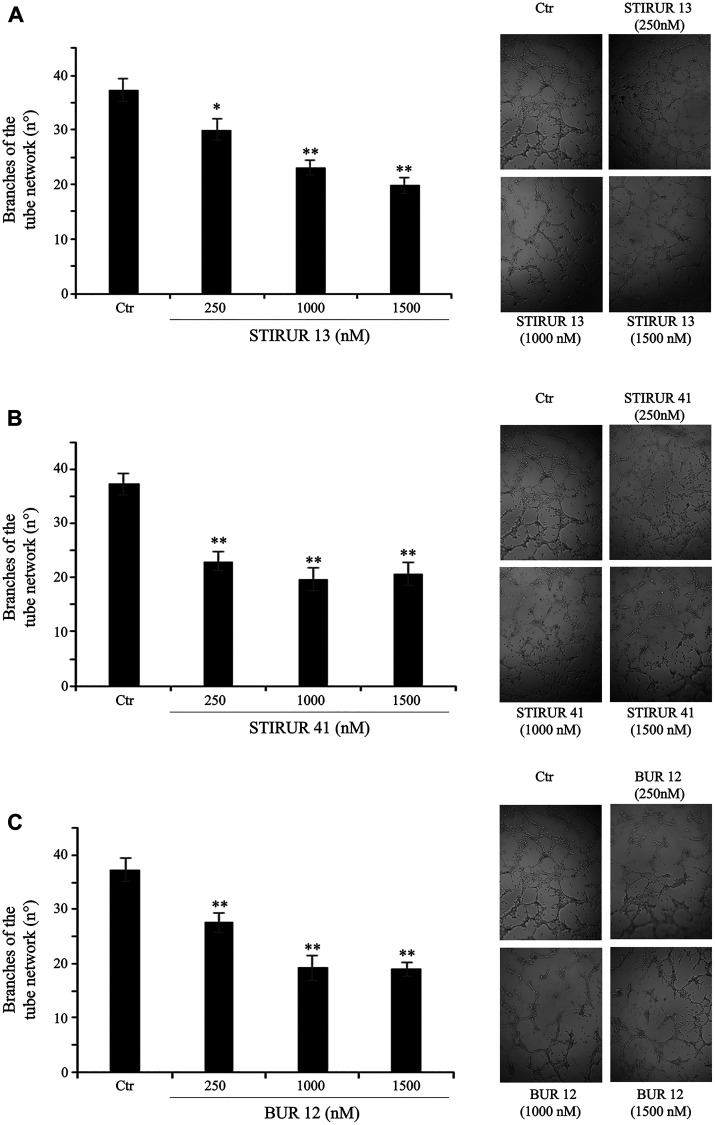
STIRUR 13, STIRUR 41 and BUR 12 reduce the ability of HTLA-230 cells to form capillary-like structures. Representative micrographs of the complete network of tubes formed by untreated (Ctr) and treated HTLA-230 cells. Cells were seeded and then treated with 250, 1000 and 1500 nM of STIRUR 13 (**A**), STIRUR 41 (**B**) or BUR 12 (**C**) for 24 h. Original magnification 10×. The graph reports the number of branches of the tube network formed by cells under the treatment conditions as above described. Quantitative data is represented by the means ± SD of three independent experiments. ^**^ *p* < 0.01 vs. control cells.

Since it has been demonstrated that HTLA-230 are able to form capillary-like structure [5] and that the expression and secretion of IL-8 [22, 24] is crucially involved in vascular mimicry [10, 13–15], the ability of STIRUR 13, STIRUR 41 and BUR 12 to modulate IL-8 and VEGF expression levels and the formation of capillary-like structures was tested. As shown in Figure 7A, both NB cells did not express IL-8 and the treatments with the three compounds did not induce any change. In addition, only HTLA-230 expressed VEGF and, also in this case, STIRUR 13, STIRUR 41 and BUR 12 treatments did not induce alterations of VEGF basal expression level (Figure 7B). Based on this finding, the analysis of vascular mimicry was performed only in HTLA-230 cells treated with STIRUR 13, STIRUR 41 and BUR 12 for 24 h. Interestingly, STIRUR 41 had the major inhibitory effect: in fact, at the lowest dose tested (250 nM), it had already decreased the number of branches in the tube network by 40% in respect to the control (Figure 8A), while STIRUR 13 and BUR 12, at the same dose of 250 nM, reduced the branches by only 20% and 25%, respectively (Figure 8B and 8C). At the highest doses employed, all the compounds similarly affected the number of branches leading to 45–50% inhibition (Figure 8). This data demonstrated that STIRUR 13, STIRUR 41 and BUR 12 were all able to inhibit the capacity of NB cells to form vascular-like structures and also supported the notion that pyrazolyl and dihydro-imidazo-pyrazolyl-urea derivatives could be employed to counteract the formation of tumor-derived blood vessels.

## DISCUSSION

A close relationship between neutrophil infiltration in neoplastic tissues and the ability of cancer cells to metastasize has been widely demonstrated in literature [25, 26]. Interestingly, the presence of neutrophils in cancers has a contradictory role since, on one hand, it induces cancer cell death and, on the other, it can favor cancer cell proliferation [27, 28]. Moreover, cytokine-mediated molecular talk between neoplastic and stromal cells is involved in reducing the efficacy of the immune response against cancer [29–31] and it has been observed that organs, such as bone marrow, liver, lung and intestine, release several chemokines able to attract cancer cells, thus facilitating the tumor spread [32–34].

Since pyrazolyl-urea and dihydro-imidazo-pyrazolyl-urea derivatives have been previously shown to reduce neutrophil migration *via* the p38MAPK-dependent pathway [1–4], the aim of this study was to investigate their ability to influence neuroblastoma survival and migration which are demonstrated to be modulated by p38MAPK inhibitors [5, 7]. In this context, NB cell lines, which were selected to perform the biological assays, are characterized by a different status of MYCN-amplification that has been found to correlate with tumor aggressiveness, increased metastatic potential, poor prognosis and resistance to therapy [35, 36] remaining the most widely accepted predictive parameter of long-term, disease-free survival in the clinical setting [37]. Furthermore, it has been demonstrated that the presence of inflammatory cells in MYCN-amplified neuroblastomas provides a validated signature of poor prognosis and the increased expression of inflammation-related genes allows the identification of a subgroup of high-risk patients who may benefit from treatments targeting tumor and its microenvironment [38].

Herein, our *in vitro* results demonstrate that pyrazolyl-urea and dihydro-imidazo-pyrazolyl-urea compounds, chosen for the study, are able to limit the migration of NB cells without affecting their viability and capacity to form colonies. In detail, in our experimental context, STIRUR 13 and STIRUR 41 are more effective in reducing the migratory ability of HTLA-230 cells in comparison to their effect on ACN cells. The different response of the two cancer cell populations could be related to the different MYCN status. In fact, HTLA-230, characterized by MYCN amplification, are more “migrating” than the non-amplified ACNs and the three compounds tested are able to reduce HTLA-230 cell migration without inducing changes in cell viability and in phosho-p38MAPK/p38MAPK levels. Therefore, considering that p38MAPK inhibitors in combination with etoposide reduce viability and migration of HTLA-230 cells [5], the compounds, herein tested, are able to counteract HTLA-230 cell motility in a p38MAPK-independent manner without affecting cell viability.

Moreover, given that STIRUR 13, STIRUR 41 and BUR 12, have a strong inhibitory effect on IL-8-induced chemotaxis of human neutrophils [1–3] and that IL-8 is differently expressed in human NB [12] and is related to angiogenesis as well as vascular mimicry [12–15], the hypothesis of IL-8 involvement has been considered. Surprisingly, the analysis of IL-8 expression levels demonstrates that the ability of the compounds to induce vascular mimicry in both NB cell lines, characterized by a different MYCN amplification state, is not related to IL-8 expression.

Instead, only HTLA-230 cells, displaying MYCN amplification, express pro-angiogenic VEGF whose expression is not modified after treatment with the compounds which are able to inhibit the formation of vascular like structures.

Therefore, in our context MYCN amplification state is not related to IL-8 expression, while it seems related to VEGF expression which in turn is not involved in the antiangiogenic activity of the compounds tested.

**Figure 9:**
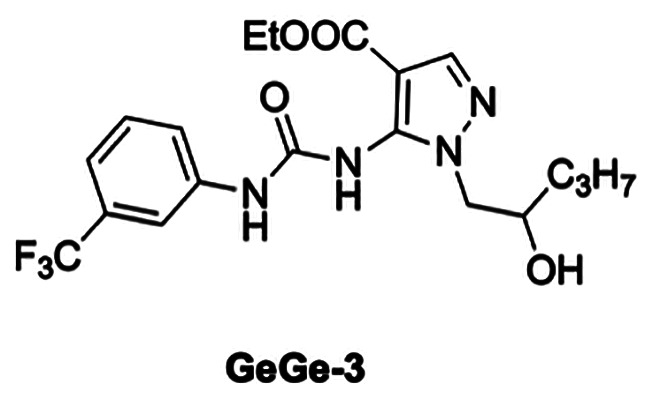
Chemical structure of previous anti-angiogenic compound GeGe3.

Our *in vitro* results, taken as a whole, suggest that pyrazolyl-urea and dihydro-imidazo-pyrazolyl-urea derivatives, which have been previously demonstrated to reduce neutrophil migration, could be potential anti-cancer agents. Indeed, STIRUR 41 is capable of inhibiting NB vascular mimicry and these findings are in line with our recent study demonstrating that GeGe3, another pyrazolyl-urea derivative [39, 40], with a similar structure to STIRUR41 (Figure 9), markedly inhibited physiological and tumor angiogenesis, *in vitro* and *in vivo*, by directly targeting MAPK and PI3K pathways and dystrophia myotonica protein kinase (DMPK)1 [39, 40].

This study represents the starting point to design new pyrazolyl-urea and dihydro-imidazo-pyrazolyl-urea derivative molecules able to fight neuroblastoma malignancy thus counteracting cell migration and vascular mimicry, both of which represent important hallmarks responsible for cancer growth and progression [41]. Furthermore, since it has been reported that pyrazole derivatives can decrease the activity of inducible isoform of Nitric Oxide Synthases (iNOS) [42], the molecules could exert their biological effect *via* iNOS modulation suggesting a potential area of investigation for the next future. However, considering their ability to inhibit neutrophil chemiotaxis these compounds-might be tested *in vivo* to block the infiltration of inflammatory cells, present in the tumoral microenvironment, which can contribute to support cancer survival [38, 41]. Noteworthy, *in vivo* studies could be important to exclude the presence of active metabolites potentially generated from the carboxyethyl ester and carboxyamide functions present in the heterocycle scaffold.

Therefore, further investigations are needed to investigate the molecular mechanisms underlying the biological effects of these compounds in order to fight the progression of neuroblastoma in high-risk patients with poor prognosis.

## MATERIALS AND METHODS

### Cell cultures and treatment

Stage-IV human neuroblastoma (NB) cell lines HTLA-230 (MYCN-amplified) and ACN (without MYCN amplification) were obtained from Lizzia Raffaghello (G. Gaslini Institute, Genoa, Italy). The cells were tested for mycoplasma contamination (Mycoplasma PCR Detection, ABM Inc., Richmond, Canada). After thawing and eight passages in the culture, cell morphology and proliferation were analyzed. NB cells were cultured in RPMI 1640 (Euroclone spa, Pavia, Italy) supplemented with 10% fetal bovine serum (FBS; Euroclone), 2 mM glutamine (Euroclone), 1% penicillin/streptomycin (Euroclone), 1% sodium pyruvate (Sigma-Aldrich Co., Saint Louis MO USA), and 1% of aminoacid solution (Sigma). Cells were treated with STIRUR 13, STIRUR 41 and BUR 12 for 24 h with doses ranging from 250 nM to 1500 nM. In some experiments, NB cells were treated with STIRUR 13, STIRUR 41 and BUR 12 at the highest dose tested (1500 nM) for 19 h and then co-treated with 500 μM H_2_O_2_ up to 24 h.

The stock solutions of the three compounds were prepared in DMSO (Sigma) and pilot experiments demonstrated that the final DMSO concentrations did not change any cell response analyzed.

### MTT assay

Cell viability was evaluated as previously described [43] by using the dimethylthiazolyl-2-5-diphenyltetrazolium bromide (MTT, Sigma) staining. Briefly, cells were seeded (90 × 10^3^ cells/well) in 96-well plates (Corning Incorporate, NY USA) and then treated with STIRUR 13, STIRUR 41 and BUR 12 for 24 h. Next, the cells were incubated with 0.5 mg/ml MTT for 3 h at 37°C. After incubation, the supernatant was discarded, insoluble formazan precipitates were dissolved in HCl (0.1 N in isopropanol) and the absorbance at 570/630 nm was recorded using a microplate reader (EL-808, BIO-TEK Instruments Inc., Winooski, Vermont, USA).

### Clonogenic assay

Clonogenic assay was evaluated as previously reported [44]. Briefly, ACN and HTLA-230 cells (150 per well) were seeded in 6-well plates (Corning) and treated with STIRUR 13, STIRUR 41 and BUR 12 for 24 h. Subsequently, the medium was changed and the cells were maintained in molecule-free medium for 20 days. ACN cells proved incapable of forming colonies by this method, and only HTLA-230 cells were then fixed with methanol and stained with crystal violet (0.5% in water with 50% methanol). Only colonies containing more than 30 cells were considered and the images were acquired with a Nikon Coolpix L22 camera (NIKON Corporation, Tokyo, Japan).

### Soft-agar colony formation assay

ACN cells were plated in 6-well plates and treated with STIRUR 13, STIRUR 41 and BUR 12 for 24 h. Anchorage-independent growth was carried out as follows: base agar (0.5% agar, RPMI 1640 and 10% FBS) was added to each well and allowed to solidify, and then an equal volume of top agar (0.35% agar, RPMI 1640 and 10% FBS), containing untreated or treated cells (10^3^ cells per cm^2^), was added to each well. Plates were incubated in a 5% CO_2_ humidified incubator at 37°C for 25 days. Colonies were stained with 0.005% crystal violet. Colonies containing more than 25 cells were counted by a Leica DMIRB microscope (Leica, Wetzlar, Germany) and the images were acquired with a Nikon Coolpix L22 camera (Nikon).

### Scratch assay

NB cells were plated into 24 wells (30 × 10^4^ HTLA-230 cells/well and 25 × 10^4^ ACN cells/well) and cultured until confluent in serum-free medium. A 200 μl pipette tip was used to ‘scratch’ cell monolayers and then, STIRUR 13, STIRUR 41 and BUR 12 were added for 24 h. Photomicrographs were taken using an inverted microscope (Leica DMIRB) equipped with a 10 × objective lens. In order to evaluate the cell migration rate, images were recorded at time 0, 24, and 48 h after the treatments. The distance between the two margins of the wound (rate of repair) was analyzed by Adobe Photoshop 7.0.1 and represents an indirect method to measure cell migration.

### Migration assay

Cell migration assay was carried out using the Transwell system (Corning) equipped with 8-mm pore size polycarbonate filters. Cells (5 × 10^4^), suspended in serum-free medium, were plated into the upper chambers in the presence or absence of STIRUR 13, STIRUR 41 or BUR 12 and allowed to migrate towards the lower chamber containing 5% FBS medium, as a chemoattractant, for 24 h. Subsequently, the non-migrating cells in the upper compartment were removed using cotton swabs and the migrating cells in the lower surface of the filters were fixed with glutaraldehyde 2.5% (Sigma) and stained with Gill’s hematoxylin no.1 solution (Sigma), according to manufacturer’s instructions. The quantity of cells that had migrated through the filter was evaluated by microscopy analysis (Leica DMIRB microscope) using a 10× objective lens.

### Immunoblot analysis

Immunoblots were carried out as previously reported [44] using polyclonal rabbit antibodies, anti-human anti-p38MAPK, anti-phospho-p38MAPK (Cell Signalling Technology Inc., Danvers, MA, USA), anti-IL8 (ThermoFisher Scientific, Rockford, USA), anti GAPDH (Santa Cruz Biotechnology, Inc, Texas, USA) and mouse antibody anti-VEGF (Santa Cruz Biotechnology).

### Formation of capillary-like structures

*In vitro* formation of capillary-like structures was carried out, as previously reported (5), on untreated and treated HTLA-230 cells (2 × 10^4^ cells/well), seeded in a 96-Matrigel-coated well plate, adding Endothelial Basal Medium in the presence or absence of VEGF (15 ng/ml) and bFGF (50 ng/ml). Samples were analyzed over a 4–48 h period with a microscope (Leica DMIRB) using 10× and 20× objective lenses.

### Data analysis

Results were expressed as mean ± SEM from at least three independent experiments. The statistical significance of parametric differences among the sets of experimental data was evaluated by one-way ANOVA and Dunnett’s test for multiple comparisons.

## Abbreviations

NB: Neuroblastoma
PI3K: phosphatidylinositol 3-kinase
phospholipase C, MAPKs: mitogen-activated protein kinases
ERK1/2: extracellular response kinases 1 and 2
IL-8: interleukin-8
fMLP: N-formyl-methionyl-leucyl-phenylalanine
PKC: protein kinase C
(PKB)/Akt: protein kinase B
MIF: migration inhibitory factor
MTT: dimethylthiazolyl-2-5-diphenyltetrazolium bromide
