# Correction: Biological evaluation of pyrazolyl-urea and dihydro-imidazo-pyrazolyl-urea derivatives as potential anti-angiogenetic agents in the treatment of neuroblastoma

**DOI:** 10.18632/oncotarget.28346

**Published:** 2023-02-11

**Authors:** Barbara Marengo, Elda Meta, Chiara Brullo, Chiara De Ciucis, Renata Colla, Andrea Speciale, Ombretta Garbarino, Olga Bruno, Cinzia Domenicotti

**Affiliations:** ^1^Department of Experimental Medicine, General Pathology Section, University of Genoa, Genoa, Italy; ^2^Department of Pharmacy, Medicinal Chemistry Section, University of Genoa, Genoa, Italy; ^3^Department of Oncology, Laboratory of Angiogenesis and Vascular Metabolism, Katholieke Universiteit Leuven, Leuven, Belgium; ^4^Laboratory of Angiogenesis and Vascular Metabolism, Center for Cancer Biology, Vlaams Instituut voor Biotechnologie, Leuven, Belgium; ^5^UOC Mutagenesis and Cancer Prevention, IRCCS Ospedale Policlinico San Martino, Genoa, Italy; ^*^These authors contributed equally to this work

**This article has been corrected:** In Figure 4C, the 3rd panel in the ‘48 h’ row under the ‘HTLA-230’ column heading is an accidental duplicate of the 4th panel of the ‘48 h’ row under the same column in 4B. The corrected Figure 4 is shown below. In addition, the figure legends shown below have been updated to clarify that the images shown are from one experiment using the single control for all three treatments. The new text is as follows:

**Figure 2: STIRUR 13, STIRUR 41 and BUR 12 did not affect cell viability of ACN and HTLA-230 cells.** Cell viability was determined by MTT assays. ACN cells (left panels) and HTLA-230 (right panels) cells were exposed to increasing concentrations (250, 1000 and 1500 nM) of STIRUR 13 (**A**) or STIRUR 41 (**B**) or BUR 12 (**C**) for 24 h. Untreated ACN or HTLA-230 cells (Ctr) were used to compare the effects of all treatments vs. control conditions. Histograms summarize quantitative data of the means ± S. E. M. of four independent experiments.

**Figure 3: STIRUR 13, STIRUR 41 and BUR 12 did not affect the clonogenic potential of ACN and HTLA-230 cells.** The clonogenic potential of ACN (left panels) and HTLA-230 (right panels) cells was evaluated by soft-agar colony formation assay and clonogenic assay, respectively. ACN cells were seeded in 6-well plates and then treated with 250, 1000 and 1500 nM of STIRUR 13 (**A**), STIRUR 41 (**B**) or BUR 12 (**C**) for 24 h, washed and re-plated in agar-containing medium. After 25 days, colonies were stained and counted. HTLA-230-cells were seeded in 6-well plates and then incubated with 250, 1000 and 1500 nM of STIRUR 13 (A), STIRUR 41(B) or BUR 12 (C) for 24 h. Subsequently, cells were incubated in fresh medium without the drug for an additional 20 days before staining and counting the colonies. Untreated ACN or HTLA-230 cells (Ctr) were used to compare the effects of all treatments vs. control conditions. Histograms summarize quantitative data of the means ± S. E. M. of four independent experiments.

**Figure 5: Effects of STIRUR 13, STIRUR 41 and BUR 12 on the migration of ACN and HTLA-230 cells.** Cell migration was evaluated by the Transwell assay and quantified by counting the number of cells which moved to the underside of the membrane after 24 h of treatment. ACN (left panels) and HTLA-230 (right panels) cells were seeded in 24-well plates and then treated with 250, 1000 and 1500 nM of STIRUR 13 (**A**), STIRUR 41 (**B**) or BUR 12 (**C**) for 24 h. Untreated ACN or HTLA-230 cells (Ctr) were used to compare the effects of all treatments vs. control conditions. Histograms summarize quantitative data of means ± S. D. of three independent experiments. ^*^ *p* < 0.05 vs. control cells; ^**^ *p* < 0.01 vs. control cells.

**Figure 8: STIRUR 13, STIRUR 41 and BUR 12 reduce the ability of HTLA-230 cells to form capillary-like structures.** Representative micrographs of the complete network of tubes formed by untreated (Ctr) and treated HTLA-230 cells. Cells were seeded and then treated with 250, 1000 and 1500 nM of STIRUR 13 (**A**), STIRUR 41 (**B**) or BUR 12 (**C**) for 24 h. Original magnification 10×. The graph reports the number of branches of the tube network formed by cells under the treatment conditions as above described. Untreated ACN or HTLA-230 cells (Ctr) were used to compare the effects of all treatments vs. control conditions. The same images of Ctr conditions were reported for all treatments for facilitating the comparison between untreated and treated cells. Quantitative data is represented by the means ± SD of three independent experiments. ^**^ *p* < 0.01 vs. control cells.

Original article: Oncotarget. 2020; 11:3459–3472. 3459-3472. https://doi.org/10.18632/oncotarget.27733

**Figure 4:**
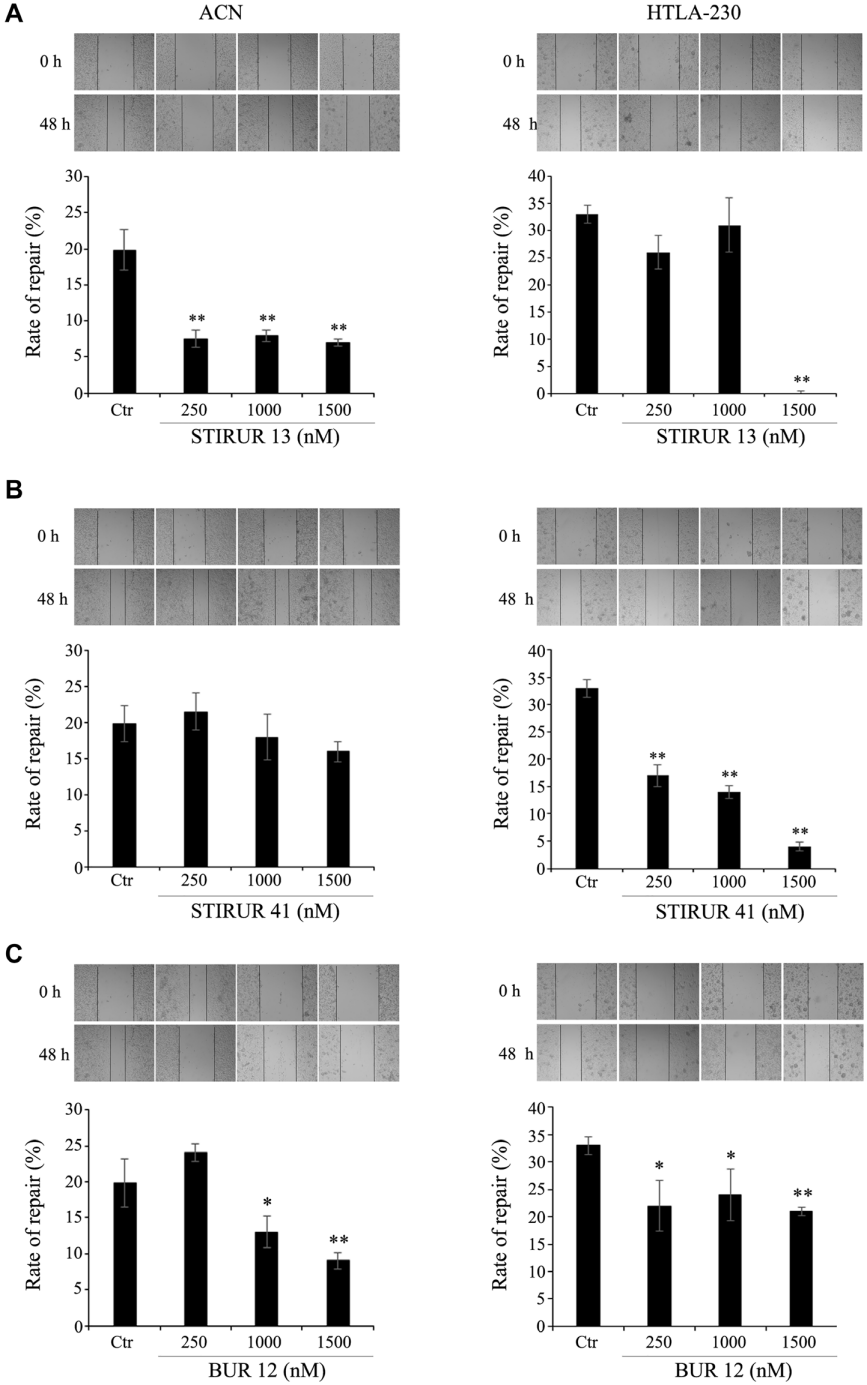
STIRUR 13, STIRUR 41 and BUR 12 affect the scratch repair ability of ACN and HTLA-230 cells. The ability of cells to heal the wound was evaluated by the scratch assay. Representative images of ACN (left panels) and HTLA-230 cells (right panels) scratch assays. The images were taken immediately after the scratch formation and after 48 h. Black lines indicate the wound borders at the beginning of the assay (0 h) and after 48 h. The rate of repair, an indirect method to measure cell migration, was quantified by measuring the distance between the migrating cell boundaries. ACN (left panels) and HTLA-230 (right panels) cells were seeded in 24-well plates and then treated with 250, 1000 and 1500 nM of STIRUR 13 (**A**), STIRUR 41 (**B**) or BUR 12 (**C**) for 24 h. Untreated ACN or HTLA-230 cells (Ctr) were used to compare the effects of all treatments vs. control conditions. The same images of Ctr conditions were reported for all treatments for facilitating the comparison between untreated and treated cells. Histograms summarize quantitative data of means ± S. D. of three independent experiments. ^*^ *p* < 0.05 vs. control cells; ^**^ *p* < 0.01 vs. control cells.

